# Multiple sclerosis: an example of pathogenic viral interaction?

**DOI:** 10.1186/s12985-017-0719-3

**Published:** 2017-02-28

**Authors:** Walter Fierz

**Affiliations:** labormedizinisches zentrum Dr Risch, Landstr. 157, 9494 Schaan, Fürstentum Liechtenstein

**Keywords:** Multiple Sclerosis, HHV-6A, EBV, EBNA-2, LMP1, MHC2TA, RBPJ-kappa, Syncytin-1, HERV-K18

## Abstract

A hypothesis is formulated on viral interaction between HHV-6A and EBV as a pathogenic mechanism in Multiple Sclerosis (MS). Evidence of molecular and genetic mechanisms suggests a link between HHV-6A infection and EBV activation in the brain of MS patients leading to intrathecal B-cell transformation. Consequent T-cell immune response against the EBV-infected cells is postulated as a pathogenic basis for inflammatory lesion formation in the brain of susceptible individuals. A further link between HHV-6A and EBV involves their induction of expression of the human endogenous retrovirus HERV-K18-encoded superantigen. Such virally induced T-cell responses might secondarily also lead to local autoimmune phenomena. Finally, research recommendations are formulated for substantiating the hypothesis on several levels: epidemiologically, genetically, and viral expression in the brain.

## Background

The role of viral interactions in pathogenesis has been reported for several human diseases. Examples are coinfection with HCV and HIV [1] or other hepatitis viruses [2] as well as with HIV and HHV-6 [3] or HHV-7 [4].

For a long time a viral aetiology of Multiple Sclerosis (MS) has been suspected und discussed. Evidence for it has often been circumstantial and sometimes not reproducible. However, in the past 20 to 30 years the importance of two viruses for MS has stood the test of time and recent reviews have summarized the evidence for their aetiological role in MS – Human Herpes Virus 6A (HHV-6A) [5] and Epstein Barr Virus (EBV) [6, 7]. The hypothesis on a viral interaction between HHV-6A and EBV as a pathogenic mechanism in multiple sclerosis (MS) has first been formulated by the author in 2004 [8]. However, a possible connection between the two herpes viruses has not been further studied in detail so far. Here, it will be argued that that the involvement of two different herpes viruses in MS is not circumstantial but fundamental to the aetio-pathogenetic processes in MS. This view could partly explain why two highly prevalent viruses are causing a relatively rare disease. First, some of the pertinent evidence for the involvement of the two viruses in MS will be summarized, and then the pieces of the puzzles will be put together in sight of new evidence supporting the picture.

## HHV-6A

An important fact is that all evidence relating MS to HHV-6 involves HHV-6A but not HHV-6B, two quite different herpes viruses [9]. Leibovitch & Jacobson (2014) have recently reviewed the role of HHV-6A in MS [5].

The basic concept is that HHV-6A is a neurotropic virus infecting the astrocytes of MS-patients [10]. The active replication of HHV-6A in MS patients correlates with a polymorphism of *MHC2TA* (rs4774C) [11], a gene that has been associated with MS [12]. *MHC2TA* is coding for the MHC class II transactivator (CIITA) which plays an essential role in the expression of MHC class II molecules by astrocytes [13]. MHC class II molecules are essential for presentation of antigens to CD4-T-cells, although IFN-γ is needed as an additional factor to enable astrocytes to do so [14, 15]. There seems also to be a combined effect of IFN-γ and HHV-6A infection on astrocytes resulting in the upregulation of ICSBP (IRF8) in astrocytes [16], a gene that has been associated with MS [17]. The HHV-6A replication related *MHC2TA* rs4774 minor allele (C) seems to diminish the expression of MHC class II molecules and therefore might allow the immune escape of the virus, similarly as described for CMV in an astrocytoma cell line [18]. Nevertheless, oligoclonal IgG from MS patients, specific for the HHV-6-A major capsid proteins have been demonstrated [19], indicating that viral immune escape is not absolute.

## EBV

The role of EBV in MS has recently been reviewed by Pender (2011) [6] and Owens & Bennett (2012) [7]. Here, the basic concept is that B-cells in the brain of MS-patients are EBV-transformed, and that the cellular immune response towards the EBV-infected cells is the hallmark of the inflammatory basis of MS. Intrathecal CD8 T-cells of MS patients recognizing lytic Epstein-Barr virus proteins have been described recently [20, 21].

Indirect evidence for transformation of B-cells in the CNS of MS patients comes from early observations that the pattern of oligoclonal bands in the CSF of MS patients is stable over time [22]. This patient-specific antibody “fingerprint” has recently been analysed at the level of epitope specificities that stayed identical over several years [23]. A systemic examination of the specificities of such antibodies produced by B-cells in the CNS of MS patients revealed no MS-specific pattern [24]. One explanation for these findings would be that such specificities are random as one would expect from EBV-transformed and immortalized B-cells [25].

EBV persistence has been described in post-mortem brain tissue of MS patients and viral reactivation has been localized to acute lesions and ectopic B-cell follicles in the meninges [26]. Notably, EBNA2+ and LMP1+ cells were detected in perivascular inflammatory cell infiltrates in active white matter lesions. However, others could not reproduce such direct evidence of EBV infection in brains of MS patients and the issue still is controversial (reviewed in [27]). On the other hand, oligoclonal IgG from MS patients, specific for the EBV proteins BRRF2 and EBNA-1 could be demonstrated [28, 29].

## The link

The basic concept is that HHV-6A actives latent EBV in B-cells resident in MS lesions.

The activation of EBV in B-cells by HHV-6 has first been reported 1993 by Louis Flamand et al [30]. In 1995 Laura Cuomo et al. [31] demonstrated that such activation of EBV is only a property of HHV-6A but not HHV-6B. Later Laura Cuomo et al. [32] have shown that HHV-6A not only induces EBV-encoded proteins associated with the lytic cycle but also upregulates expression of EBV-encoded growth-transformation-associated proteins, namely LMP1 and EBNA-2.

The activation of EBNA-2 expression by HHV-6A deserves a special interest since recently it has been found that the association between EBV and MS is dependent on genetic variants of EBV involving particularly the *EBNA2* gene, the most polymorphic region of the viral genome [33].

This genetic link between *EBNA2* polymorphism and MS again deserves further interest since EBNA-2 directly interacts with the cellular DNA-binding protein RBPJ-κ (recombination signal-binding protein J kappa), a ubiquitous protein of the Notch signalling pathway that plays an important role in Epstein-Barr virus infection. Notch signalling may play a role in MS pathogenesis by affecting function of both immune and glial cells (reviewed in [34]).

EBNA-2 regulation of transcription through RBPJ-κ is essential for resting B-lymphocyte conversion to immortal B-cells [35, 36]. Interestingly, RBPJ is a possible autoantigen in MS recognized by CSF-derived immunoglobulin G in a subset of patients with MS [37]. On the molecular level, therefore, the link between HHV-6A and EBV can be pinned down to two interacting proteins: HHV-6A induced EBNA-2 and cellular host protein RBPJ-κ. Such physical interaction between a foreign (viral) protein and a self-protein might well turn the RBPJ-κ into an autoantigen, similarly as it is the case with gliadin and tissue transglutaminase in celiac disease.

The upregulation of LMP1 by HHV-6A in EBV infected cells [32] suggests a further interesting link to MS: As shown in EBV-related nasopharyngeal carcinoma cells, LMP1 induces an increasing expression of kappa light chains [38]. On the other hand, as known for many years, intrathecal expression of kappa light chains is a consistent finding in CSF of MS patients, having even a prognostic value [39]. A recent multicentre study has confirmed the diagnostic value of kappa free light chains in CSF as diagnostic biomarkers in MS [40]. The question remains what cells in the CNS would produce the kappa light chains. Apart from B-cells, interesting candidates are astrocytes infected with both EBV [41] and HHV-6A [10]. EBV infection of astrocytes activates the HERV-W/MSRV/syncytin-1 [41]. The HERV-W-related retroelement, MS retrovirus (MSRV), particularly its envelope protein syncytin-1, has been reported to be overexpressed in white matter lesions of individuals with MS (reviewed in [42]).

A further link between HHV-6A and EBV comes from the observation that both viruses induce expression of the human endogenous retrovirus HERV-K18-encoded superantigen [43, 44]. Furthermore, the *HERV-K18.3 env* genotype is a potential risk factor for MS [45]. Interestingly, the HERV-K18-encoded superantigen stimulates T-cells carrying receptors of the Vβ13 family [46] in which also T-cell clones with specificity for an immunodominant peptide of myelin basic protein (MBP) have been found in MS patients [47]. This example is demonstrating how a viral infection might lead to or enhance an autoimmune reaction.

## Disease-modifying therapies in MS

Support for a combined pathogenic role of HHV-6A and EBV in MS might come from studies involving disease-modifying therapies. In fact, the first trial in MS with specific anti-herpes virus treatment showed that acyclovir might inhibit the triggering of MS [48]. Later, the introduction of IFN-β as the first disease modifying therapy of MS was initially based on the assumption that the anti-viral effect of IFN-β is the basis of its treatment effect. Particularly, an interference with EBV and MSRV is suspected. However, the exact mode of its action is not yet fully understood (for review see [49]). Apart from EBV and MSRV, IFN-β treatment seems to affect HHV-6 expression. Patients that fail to suppress HHV-6 during IFN-β treatment have been demonstrated to also show a poor clinical response [50].

Another line of evidence supporting the pathogenic role of HHV-6A and EBV in MS comes from the treatment effect of B-cell depletion. Originally, chimeric monoclonal antibodies against the pan-B cell marker CD20, in form of rituximab, were used to successfully treat EBV-related post-transplant lymphoproliferative disorders (PTLD) [51]. The idea of B-cell depletion with rituximab was then successfully adopted to treatment of relapsing-remitting MS [52]. Even better results were later achieved by using the fully humanized anti-CD20 monoclonal antibody ocrelizumab (OCR) that showed to be effective not only in relapsing-remitting MS but also in primary progressive MS [53], illustrating the central role of B-cells in the pathogenesis of MS.

The third line of evidence comes from treatment with the α_4_ integrin (VLA-4) antagonist natalizumab. Treatment with natalizumab carries the risk of provoking progressive multifocal leukoencephalopathy (PML) caused by latent JC virus in the CNS (for review see [54]). However, in addition HHV-6 seems to be reactivated in the CNS of some natalizumab treated patients [55].

None of the above-mentioned disease-modifying therapies in MS directly prove the postulated interaction of HHV-6A and EBV, but all of them are well in line with infection of both HHV-6A and EBV in the brain of MS patients.

## Conclusion

A link is postulated between HHV-6A and EBV in the aetio-pathogenesis of MS.

In summary, the tenet is that infection with the neurotropic HHV-6A leads to transformation of latently EBV-infected B-cells in the CNS. Both viruses will elicit a T-cell response, either specific towards HHV-6A and EBV, or non-specific as a response to the HERV-K18-encoded superantigen. Such viral induced T-cell responses might secondarily also lead to autoimmune phenomena. Evidence for mechanisms for induction of autoimmunity by viral infections has recently been reviewed [56].

The hypothesis could be tested on several levels:

- Epidemiological level: The prevalence of genetic subtypes of EBV (*EBNA2*) and HHV-6A and its subtypes are not known. Establishing the prevalence of co-infection with the two viruses is crucial to estimate the likelihood of their combined effects in MS.
- Level of genetic associations to MS: The relations between *MHC2TA* as well as *EBNA2* polymorphisms with MS suggest a link between active replication of HHV-6A, regulated by *MHC2TA*, and HHV-6A-induced EBNA2 expression in EBV-infected cells. A crucial test would be to look for genetic interaction between all three polymorphisms, *MHC2TA, EBNA2*, and *HERV-K18* in the risk for MS.
- Molecular level: Both, findings for HHV-6A and EBV expression in MS lesions are not unanimous. Studies looking for co-expression of the two viruses in brain tissue of MS patients using the MALDI-TOF MS technology [57] and/or multiplex detection of herpes viruses in CSF by PCR [58] might clarify the issue.

## Abbreviations

BRRF2: Tegument protein BRRF2
CMV: Cytomegalo virus
CNS: Central nervous system
CSF: Cerebrospinal fluid
EBNA2: Epstein Barr Nuclear Antigen 2
EBV: Epstein Barr Virus
HCV: Hepatitis Virus C
HERV: Human endogenous retrovirus
HHV-6A: Human Herpes Virus – 6A
HIV: Human Immunodeficiency Virus
ICSBP, IRF8: Interferon Consensus Sequence-Binding Protein, Interferon Regulatory Factor 8
IFN-β: Interferon beta
IFN-γ: Interferon gamma
LMP1: Latent Membrane Protein 1
MALDI-TOF MS: Matrix Assisted Laser Desorption Ionization — Time of Flight Mass Spectrometry
*MHC2TA,* CIITA*,* Class II: Major Histocompatibility Complex, Transactivator Gene
MS: Multiple Sclerosis
MSRV: Multiple sclerosis retrovirus
PCR: Polymerase chain reaction
RBPJ-κ: Recombination Signal-Binding Protein J kappa

## References

[1] Winnock M, Salmon-Céron D, Dabis F, Chêne G (2004). Interaction between HIV-1 and HCV infections: towards a new entity?. J Antimicrob Chemother.

[2] Lin L, Verslype C, van Pelt JF, van Ranst M, Fevery J (2006). Viral interaction and clinical implications of coinfection of hepatitis C virus with other hepatitis viruses. Eur J Gastroenterol Hepatol.

[3] Grivel JC, Ito Y, Fagà G, Santoro F, Shaheen F, Malnati MS (2001). Suppression of CCR5- but not CXCR4-tropic HIV-1 in lymphoid tissue by human herpesvirus 6. Nat Med.

[4] Lisco A, Grivel J-C, Biancotto A, Vanpouille C, Origgi F, Malnati MS (2007). Viral interactions in human lymphoid tissue: Human herpesvirus 7 suppresses the replication of CCR5-tropic human immunodeficiency virus type 1 via CD4 modulation. J Virol.

[5] Leibovitch EC, Jacobson S (2014). Evidence linking HHV-6 with multiple sclerosis: an update. Curr Opin Virol.

[6] Pender MP (2011). The essential role of Epstein-Barr virus in the pathogenesis of multiple sclerosis. Neuroscientist.

[7] Owens GP, Bennett JL (2012). Trigger, pathogen, or bystander: the complex nexus linking Epstein- Barr virus and multiple sclerosis. Mult Scler.

[8] Fierz W. Virale Hypothese der Multiplen Sklerose. In: Kesselring J, editor. Multiple Sklerose. 4th ed. Stuttgart: Kohlhammer-Verlag; 2004.

[9] Ablashi D, Agut H, Alvarez-Lafuente R, Clark DA, Dewhurst S, DiLuca D (2014). Classification of HHV-6A and HHV-6B as distinct viruses. Arch Virol.

[10] Donati D, Martinelli E, Cassiani-ingoni R, Ahlqvist J, Hou J, Major EO (2005). Variant-specific tropism of human herpesvirus 6 in human astrocytes. J Virol.

[11] Alvarez-Lafuente R, Martinez A, Garcia-Montojo M, Mas A, De Las Heras V, Dominguez-Mozo MI (2010). MHC2TA rs4774C and HHV-6A active replication in multiple sclerosis patients. Eur J Neurol.

[12] Bronson PG, Caillier S, Ramsay PP, McCauley JL, Zuvich RL, De Jager PL (2010). CIITA variation in the presence of HLA-DRB1*1501 increases risk for multiple sclerosis. Hum Mol Genet.

[13] Dong Y, Benveniste EN (2001). Immune function of astrocytes. Glia.

[14] Fierz W, Endler B, Reske K, Wekerle H, Fontana A (1985). Astrocytes as antigen-presenting cells. I. Induction of Ia antigen expression on astrocytes by T cells via immune interferon and its effect on antigen presentation. J Immunol.

[15] Barcia C, Mitxitorena I, Carrillo-de Sauvage MA, Gallego J-M, Pérez-Vallés A (2013). Imaging the microanatomy of astrocyte-T-cell interactions in immune-mediated inflammation. Front Cell Neurosci.

[16] Meeuwsen S, Persoon-Deen C, Bsibsi M, Bajramovic JJ, Ravid R, De Bolle L (2005). Modulation of the cytokine network in human adult astrocytes by human herpesvirus-6A. J Neuroimmunol.

[17] De Jager PL, Jia X, Wang J, de Bakker PIW, Ottoboni L, Aggarwal NT (2009). Meta-analysis of genome scans and replication identify CD6, IRF8 and TNFRSF1A as new multiple sclerosis susceptibility loci. Nat Genet.

[18] Le Roy E, Mühlethaler-Mottet A, Davrinche C, Mach B, Davignon JL (1999). Escape of human cytomegalovirus from HLA-DR-restricted CD4(+) T-cell response is mediated by repression of gamma interferon-induced class II transactivator expression. J Virol.

[19] Alenda R, Álvarez-Lafuente R, Costa-Frossard L, Arroyo R, Mirete S, Álvarez-Cermeño JC (2014). Identification of the major HHV-6 antigen recognized by cerebrospinal fluid IgG in multiple sclerosis. Eur J Neurol.

[20] Lossius A, Johansen JN, Vartdal F, Robins H, Benth JŠ, Holmøy T (2014). High-throughput sequencing of TCR repertoires in multiple sclerosis reveals intrathecal enrichment of EBV-reactive CD8(+) T cells. Eur J Immunol.

[21] Van Nierop GP (2015). Intrathecal CD8 T-cells of multiple sclerosis patients recognize lytic Epstein-Barr virus proteins. Multiple Sclerosis J.

[22] Petzold A (2013). Intrathecal oligoclonal IgG synthesis in multiple sclerosis. J Neuroimmunol.

[23] Yu X, Burgoon M, Green M, Barmina O, Dennison K, Pointon T (2011). Intrathecally synthesized IgG in multiple sclerosis cerebrospinal fluid recognizes identical epitopes over time. J Neuroimmunol.

[24] Willis SN, Stathopoulos P, Chastre A, Compton SD, Hafler DA, O’Connor KC (2015). Investigating the antigen specificity of multiple sclerosis central nervous system-derived immunoglobulins. Front Immunol.

[25] Tosato G, Blaese RM, Yarchoan R (1985). Relationship between immunoglobulin production and immortalization by Epstein Barr virus. J Immunol.

[26] Serafini B, Rosicarelli B, Franciotta D, Magliozzi R, Reynolds R, Cinque P (2007). Dysregulated Epstein-Barr virus infection in the multiple sclerosis brain. J Exp Med.

[27] Lassmann H, Niedobitek G, Aloisi F, Middeldorp JM (2011). Epstein-Barr virus in the multiple sclerosis brain: a controversial issue--report on a focused workshop held in the Centre for Brain Research of the Medical University of Vienna, Austria. Brain.

[28] Cepok S, Zhou D, Srivastava R, Nessler S, Stei S, Büssow K (2005). Identification of Epstein-Barr virus proteins as putative targets of the immune response in multiple sclerosis. J Clin Invest.

[29] Pfuhl C, Oechtering J, Rasche L, Gieß RM, Behrens JR, Wakonig K (2015). Association of serum Epstein–Barr nuclear antigen-1 antibodies and intrathecal immunoglobulin synthesis in early multiple sclerosis. J Neuroimmunol.

[30] Flamand L, Stefanescu I, Ablashi DV, Menezes J (1993). Activation of the Epstein-Barr virus replicative cycle by human herpesvirus 6. J Virol.

[31] Cuomo L, Angeloni A, Zompetta C, Cirone M, Calogero A, Frati L (1995). Human herpesvirus 6 variant A, but not variant B, infects EBV-positive B lymphoid cells, activating the latent EBV genome through a BZLF-1-dependent mechanism. AIDS Res Hum Retroviruses.

[32] Cuomo L, Trivedi P, De Grazia U, Calogero A, D’Onofrio M, Yang W (1998). Upregulation of Epstein-Barr virus-encoded latent membrane protein by human herpesvirus 6 superinfection of EBV-carrying Burkitt lymphoma cells. J Med Virol.

[33] Mechelli R, Manzari C, Policano C, Annese A, Picardi E, Umeton R (2015). Epstein-Barr virus genetic variants are associated with multiple sclerosis. Neurology.

[34] Juryńczyk M, Selmaj K (2010). Notch: a new player in MS mechanisms. J Neuroimmunol.

[35] Henkel T, Ling PD, Hayward SD, Peterson MG (1994). Mediation of Epstein-Barr virus EBNA2 transactivation by recombination signal-binding protein J kappa. Science.

[36] Zhao B, Zou J, Wang H, Johannsen E, Peng C-W, Quackenbush J (2011). Epstein-Barr virus exploits intrinsic B-lymphocyte transcription programs to achieve immortal cell growth. Proc Natl Acad Sci U S A.

[37] Querol L, Clark PL, Bailey MA, Cotsapas C, Cross AH, Hafler DA (2013). Protein array-based profiling of CSF identifies RBPJ as an autoantigen in multiple sclerosis. Neurology.

[38] Liu H, Zheng H, Li M, Hu D, Tang M, Cao Y (2007). Upregulated expression of kappa light chain by Epstein-Barr virus encoded latent membrane protein 1 in nasopharyngeal carcinoma cells via NF-kappaB and AP-1 pathways. Cell Signal.

[39] Rinker JR, Trinkaus K, Cross AH (2006). Elevated CSF free kappa light chains correlate with disability prognosis in multiple sclerosis. Neurology.

[40] Presslauer S, Milosavljevic D, Huebl W, Aboulenein-Djamshidian F, Krugluger W, Deisenhammer F (2015). Validation of kappa free light chains as a diagnostic biomarker in multiple sclerosis and clinically isolated syndrome: a multicenter study. Mult Scler J.

[41] Mameli G, Poddighe L, Mei A, Uleri E, Sotgiu S, Serra C (2012). Expression and activation by Epstein Barr virus of human endogenous retroviruses-W in blood cells and astrocytes: inference for multiple sclerosis. PLoS One.

[42] Antony JM, Deslauriers AM, Bhat RK, Ellestad KK, Power C (1812). Human endogenous retroviruses and multiple sclerosis: innocent bystanders or disease determinants?. Biochim Biophys Acta.

[43] Sutkowski N, Conrad B, Thorley-Lawson DA, Huber BT (2001). Epstein-Barr virus transactivates the human endogenous retrovirus HERV- K18 that encodes a superantigen. Immunity.

[44] Tai AK, Luka J, Ablashi D, Huber BT (2009). HHV-6A infection induces expression of HERV-K18-encoded superantigen. J Clin Virol.

[45] Tai AK, O’Reilly EJ, Alroy KA, Simon KC, Munger KL, Huber BT (2008). Human endogenous retrovirus-K18 Env as a risk factor in multiple sclerosis. Mult Scler.

[46] Sutkowski N, Palkama T, Ciurli C, Sekaly RP, Thorley-Lawson DA, Huber BT (1996). An Epstein-Barr virus-associated superantigen. J Exp Med.

[47] Hong J (1999). A Common TCR V-D-J Sequence in Vβ13.1 T Cells Recognizing an Immunodominant Peptide of Myelin Basic Protein in Multiple Sclerosis. J Immunol.

[48] Lycke J, Svennerholm B, Hjelmquist E (1996). Acyclovir treatment of relapsing-remitting multiple sclerosis. A randomized, placebo-controlled, double-blind study. J Neurol.

[49] Annibali V, Mechelli R, Romano S (2015). IFN-β and multiple sclerosis: From etiology to therapy and back. Cytokine Growth Factor Rev.

[50] Garcia-Montojo M, De Las Heras V, Dominguez-Mozo M (2011). Human herpesvirus 6 and effectiveness of interferon beta 1b in multiple sclerosis patients. Eur J Neurol.

[51] Ganne V, Siddiqi N, Kamaplath B (2003). Humanized anti-CD20 monoclonal antibody (Rituximab) treatment for post-transplant lymphoproliferative disorder. Clin Transplant.

[52] Hauser SL, Waubant E, Arnold DL (2008). B-Cell Depletion with Rituximab in Relapsing–Remitting Multiple Sclerosis. N Engl J Med.

[53] Montalban X, Hemmer B, Rammohan K (2016). Efficacy and Safety of Ocrelizumab in Primary Progressive Multiple Sclerosis: Results of the Phase III Double-Blind, Placebo-Controlled ORATORIO Study (S49.001). Neurology.

[54] Warnke C, Menge T, Hartung H-P, Racke MK, Cravens PD, Bennett JL (2010). Natalizumab and progressive multifocal leukoencephalopathy. Arch Neurol.

[55] Yao K, Gagnon S, Akhyani N, Williams E, Fotheringham J, Frohman E (2008). Reactivation of human herpesvirus-6 in natalizumab treated multiple sclerosis patients. PLoS One.

[56] Sfriso P, Ghirardello A, Botsios C, Tonon M, Zen M, Bassi N (2010). Infections and autoimmunity: the multifaceted relationship. J Leukoc Biol.

[57] Sjöholm MIL, Dillner J, Carlson J (2008). Multiplex detection of human herpesviruses from archival specimens by using matrix-assisted laser desorption ionization-time of flight mass spectrometry. J Clin Microbiol.

[58] Yamamoto T, Nakamura Y (2000). A single tube PCR assay for simultaneous amplification of HSV-1/-2, VZV, CMV, HHV-6A/-6B, and EBV DNAs in cerebrospinal fluid from patients with virus-related neurological diseases. J Neurovirol.

